# Editorial: Understandings and conceptualizations of hope and how it influences engagement with sexual and reproductive health (SRH) services among adolescents in LMICs

**DOI:** 10.3389/frph.2023.1285313

**Published:** 2023-10-20

**Authors:** Natsayi Chimbindi, Thembelihle Zuma, Andrew Gibbs, Sarah Bernays, Janet Seeley

**Affiliations:** ^1^ Africa Health Research Institute, KwaZulu-Natal, South Africa; ^2^ Institute of Global Health (IGH), University College London, London, United Kingdom; ^3^ Public Health, University of KwaZulu-Natal, Durban, South Africa; ^4^ Gender and Health Research Unit, South African Medical Research Council, Pretoria, South Africa; ^5^ Department of Psychology, University of Exeter, Exeter, United Kingdom; ^6^ School of Public Health, University of Sydney, Sydney, NSW, Australia; ^7^ Department of Global Health and Development, London School of Hygiene and Tropical Medicine, London, United Kingdom

**Keywords:** hope, adolescent, young people, sexual reproductive health, health behaviour

**Editorial on the Research Topic** Understandings and conceptualizations of hope and how it influences engagement with sexual and reproductive health (SRH) services among adolescents in LMICs

Adolescents and young people (10–24 years) constitute about 1.2 billion of the world's population, with 70% of adolescents living in lower- and middle-income countries (LMIC) (1–3). It is during adolescence that many health behaviours are shaped, and young people face a range of health risks, particularly in relation to sexual and reproductive health (SRH) and access to SRH services (Mehta and Seeley) (3). In this collection, we examine adolescent SRH risk and reflect on the role of hope in framing risk behaviours. We understand “hope” as a belief that a desired positive future outcome is possible (4) mediating how individuals respond to their wider social contexts, which may, in turn, shape sexual risk behaviours, health, and, specifically, SRH outcomes for adolescents.

Much research on hope has been driven by psychologists in the global North, with individualised understandings of hope essential to overcoming adversity and fostering resilience and positively linked to health and wellness (5–7). While this provides a platform for thinking about the topic, research in the global South has suggested that simply “importing” understandings of hope does not capture local understandings in LMIC. For example, adolescent respondents in a recent study in KwaZulu-Natal, South Africa (8), thought of hope in relation to an object, person, or goal: ‘having hope in someone or a hope for something (a wish or a dream)’ (8). Hope was also seen as ‘embedded within young people's behaviours’. Those who engaged in risk behaviours such as substance misuse were considered to have “no hope” and lack future aspirations.' (8).

Understanding the link between hope and health behaviours for adolescents can inform SRH interventions that address how young people understand themselves, their risk perception, their context, and their future. Developing understandings of how hope operates dynamically in the lives of adolescents and how hope shapes SRH outcomes is important to consider.

In this collection, we have brought together quantitative and qualitative research that examine the relationship between hope and SRH-related experiences and behaviours, including how “risks” and opportunities are constructed and navigated and how this influences adolescents' engagement (i.e., awareness, access, and uptake) with SRH services, including for HIV. The collection includes a review exploring the role of agency and hope in adolescent behaviour and access to SRH services and articles on risky sexual behaviours that predispose young people to poor SRH outcomes and understanding traditional social norms and contexts that increase young women's vulnerability and affect access to SRH services.

Groenewald et al. focused on the roles that agency and hope play in the SRH of adolescents, including accessing SRH services. They view hope as the driver of agency associated with more positive outcomes in young people, including enhanced self-esteem, self-worth, and self-care agency. Given that the link between hope and agency, access, and uptake of SRH services is unclear, their review explored how these factors converge and interact to influence adolescents' SRH and access to SRH services. Their work has highlighted the importance of understanding the role of individual factors such as hope, self-efficacy, agency, and autonomy in adolescent SRH, behaviours, and access to services and how these interact with the social environment, norms, and practices that often shape young people's risks and protective behaviours and access to services in African contexts. They cited studies demonstrating cultural norms that view girls publicly expressing sexuality and talking about sex as taboo.

Maruwo et al. aimed to identify factors associated with long-acting (LARC) and short-acting (SARC) reversible contraceptive use among 10–24-year olds in Lilongwe, Malawi. Their study analysed data of clients accessing family planning services from 64 youth outreach sites in Lilongwe district. This study has highlighted the high unmet contraception needs among young people, especially acute among younger compared to older clients, and its findings are essential to address the complex burden prompted by unplanned pregnancies. Furthermore, LARC uptake was found to vary by age, education, number of living children, and marital status, among other factors, underlining the importance of individual social contextual factors in shaping access to and uptake of preventive opportunities.

The third article by Workye et al. focused on gender-based violence (GBV), which is a key risk factor for poor SRH and influencing SRH services uptake. The study found that the overall prevalence of GBV among women university students in southwest Ethiopia was very high, with almost half of the students reporting experiencing GBV, mostly physical and sexual violence. Students who were married or living with a man, who consumed alcohol, and who did not discuss SRH issues openly with their families were more likely to experience GBV, highlighting the role of locally specific social norms in shaping risk behaviours and the need for tailored SRH for youth that responds to these conditions in any context.

Lastly, Muraguri et al. reported on the finding of a bio-behavioural survey of male sex workers (MSWs) who have sex with other men in Nairobi, Kenya, utilizing a respondent-driven sampling recruitment approach. They estimated the prevalence of HIV and STIs and documented high levels of alcohol and drug use, risky sexual practices, and experiences of violence and discrimination. Although they found both younger (18–24 years) and older (>25 years) MSWs were engaged in high-risk sexual behaviours, older MSWs were more likely to be living with HIV. Although overall rates of violence and discrimination were low, a higher proportion of younger MSWs reported verbal, physical, and sexual violence than older MSWs. These findings suggest the importance of understanding the interplay of risks across the life course for specific groups and the public health imperative of ensuring that preventive services are accessible, for example, through the provision of safe clinical spaces, even in settings where homosexuality is criminalised and where there are few or no tailored services for key populations.

Taken together, these four articles make an important contribution to the understanding of how hope and context shape adolescents' experiences, risk behaviours, and uptake of SRH services and outcomes in LMIC settings. Understanding how “risks” and opportunities are constructed and navigated is crucial for informing the development and scale-up of SRH interventions to improve SRH uptake and outcomes among adolescents in LMIC settings.
